# An Unusual Clinical Presentation of Merkel Cell Carcinoma: A Case Report

**DOI:** 10.1155/2010/905414

**Published:** 2010-03-15

**Authors:** Deba P. Sarma, Dawn E. Heagley, Julianne Chalupa, Meredith Cox, James M. Shehan

**Affiliations:** ^1^Department of Pathology, Creighton University Medical Center (DPS, DEH, JC, MC), Omaha, NE 68131, USA; ^2^Department of Dermatology (JMS), Bergan Mercy Medical Center, Omaha, NE 68124, USA

## Abstract

*Introduction*. Merkel cell carcinoma is a rare, aggressive neuroendocrine cell carcinoma arising in the sun-exposed skin of elderly patients. Most of these tumors are located in the dermis. An unusual clinical presentation of such a tumor in the subcutis, if not biopsied, may be easily mistaken as a benign lesion. *Case Presentation*. An 83-year-old white woman presented with a several-month history of a painless 7 mm subcutaneous mass that was initially thought to be a lipoma. A conservative follow-up was planned. At the insistence of the patient, an excisional biopsy of the mass was performed revealing a subcutaneous Merkel cell carcinoma. The tumor cells stained positively for CK 20, chromogranin, and synaptophysin. No other primary or metastatic tumors found after a thorough work-up. The patient was treated with local irradiation. She remains disease free at her six-month follow-up visit. *Conclusion*. When a new growth is encountered in the sun-exposed skin of elderly patients, a biopsy is warranted even if the lesion clinically appears benign.

## 1. Case Presentation

An 83-year-old white American woman of European descent presented to the Dermatology clinic complaining of a small painless “lump” on her left cheek that had been present for several months. She did not have a past history of any significant medical illness including any neoplastic disease.

Physical examination revealed a 7 mm soft papule with overlying intact skin on the left cheek. There was no evidence of any sun-induced skin lesion. There were no palpable lymph nodes in the neck. Clinical impression was that of a benign subcutaneous nodule such as a lipoma. The patient was advised of conservative follow-up, but the patient insisted on having the lesion removed. 

An excisional biopsy was performed. The excised specimen was an ovoid fragment of adipose tissue measuring 1 cm in size attached to an ellipse of normal skin measuring 1.9 × 0.5 cm.

**Figure 1:**
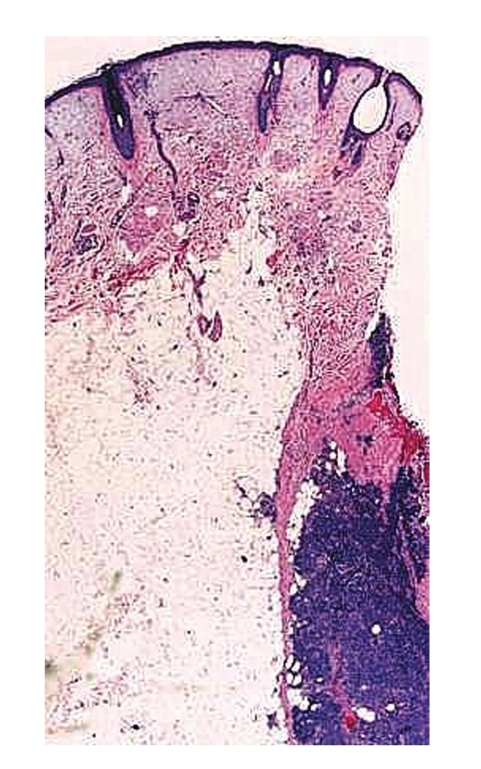
Dermal solar degeneration and dark tumor nodule in the subcutaneous tissue.

**Figure 2:**
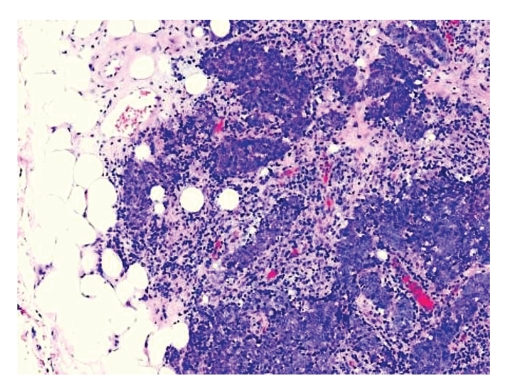
Undifferentiated dark epitheliod neoplastic infiltrates within the adipose tissue.

**Figure 3:**
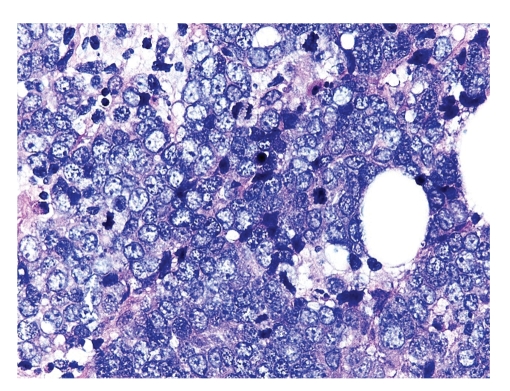
The neoplastic cells show vague cell borders, large pleomorphic nuclei, and numerous mitotic figures.

**Figure 4:**
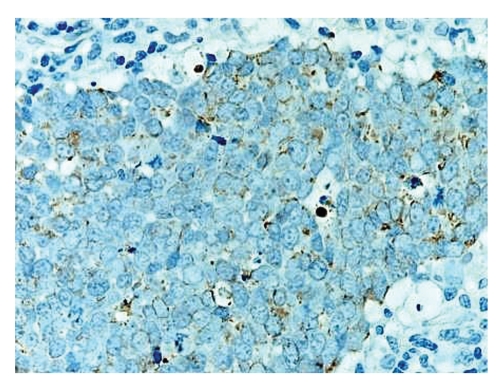
The tumor cells are positive for CK20 and show a “dot-like” pattern of staining.

**Figure 5:**
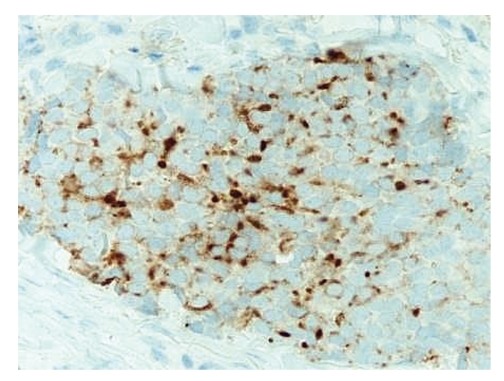
The tumor cells are positive for chromogranin.

**Figure 6:**
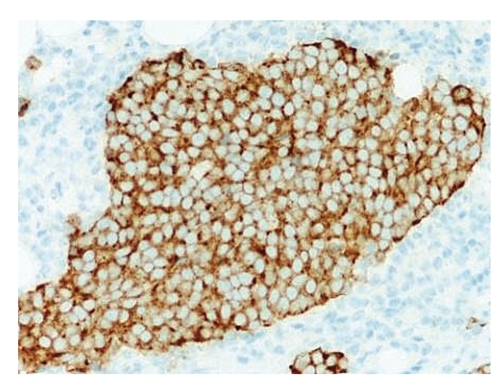
The tumor cells are positive for synaptophysin.

Microscopic examination revealed an intact atrophic epidermis, solar elastosis of the dermis, and several dark tumor nodules in the subcutaneous adipose tissue (Figures 1 and 2). The neoplasm was composed of dark, small undifferentiated cells with ill-defined cell borders showing large hyperchromatic nuclei with numerous mitotic figures (Figure 3). The tumor cells stained positively for CK 20 showing a “dot-like” pattern (Figure 4) and were also positive for chromogranin (Figure 5) and synaptophysin (Figure 6). They were negative for CK 5/6, S-100, and LCA immunostains. The diagnosis of subcutaneous Merkel cell carcinoma (MCC) was made.

A thorough clinical and radiological workup was undertaken to exclude other possible primary neuroendocrine carcinomas, especially that of lung. The patient was found to be free of other primary or metastatic tumors.

The patient refused surgical intervention and was treated with local irradiation at the biopsy site. At the six-month follow-up visit, the patient remains asymptomatic and free of tumor.

## 2. Discussion

Merkel cell carcinoma (MCC) is a rare, aggressive cutaneous carcinoma arising from neuroendocrine cells of the dermis. Clinically, MCC is found in sun-exposed areas such as the face or neck and presents as a small (less than 2 cm), red or violet raised nodule with intact overlying skin. The lesion is usually painless and rapidly growing. The populations most commonly affected are Caucasians over the age of 70 and the immunosuppressed [1–4].

Microscopically, the tumor nodule of MCC is comprised of small cells with scant cytoplasm, large nuclei, finely granular chromatin, and numerous mitotic figures. MCC is best differentiated from other cutaneous tumors by immunohistochemical stains. MCC cells stain positively for cytokeratin 20 in a characteristic “dot-like” pattern and stain negatively for S-100 (positive in melanoma), TTF-1 and CK7, (positive in small cell lung carcinoma), and LCA (positive in lymphoma) [4]. Additionally, MCC cells stain positively for neuroendocrine markers chromogranin and synaptophysin. Electron microscopy shows characteristic intracytoplasmic neurosecretory granules [1].

Merkel cell carcinoma almost always arises in the dermis, but there has been one reported case in the literature of MCC arising in subcutaneous fat [2]. Our case represents the second reported case, to our knowledge, of MCC arising in subcutaneous adipose tissue. 

Treatment of MCC includes wide excision, sentinel lymph node biopsy, and local radiation. Neoadjuvant chemotherapy is not recommended. Prognosis is variable and most dependent on stage of disease at presentation. Overall, local recurrence after excision occurs in about 40 percent of cases, while regional lymph node metastasis occurs in about 30 percent of patients [4].

Identified risk factors associated with MCC are: age greater than 50 years, European ancestry, ultraviolet (UV) radiation, immunocompromised state, or infection with Merkel Cell Polyomavirus (MCPyV). Frequency of MCC increases dramatically in patients after the age of 50 years suggesting an accumulation of oncogenic events. Regarding UV radiation, MCC tumors are present in sun-exposed areas such as the head and neck. Patients who have undergone prior psoralen and PUVA treatment are at higher incidence. Those of European decent are genetically more susceptible to this radiation and therefore have an increased prevalence of MCC. An association is also noted in patients who are immunocompromised; an 11-fold increase in incidence of MCC has been seen in patients with AIDS and a 5-fold increase in patients who have undergone organ transplantation. Finally, a correlation between MCPyV and MCC has been established [5, 6].

Recent research has strengthened the correlation between MCPyV and MCC. MCPyV DNA is found in the majority of MCC tumors but is also commonly found in normal tissue including saliva and tissues of the upper digestive tract. A recent study suggests that the MCPyV integrates into the Large T antigen prior to clonal expansion, and this interruption of the Large T antigen is not seen in nontumor cells [5]. Another study indicates that lower mortality of MCC is associated with MCPyV viral integration versus those MCCs without MCPyV [6]. This data suggests the possibility of different mechanisms for inducing oncogenesis. MCPyV DNA is ubiquitous in nature and MCC is relatively rare suggesting that this may be one of many cofactors involved in the oncogenic transformation to MCC.

## 3. Conclusion

When a new growth is encountered in the sun-exposed skin of elderly patients, a biopsy is warranted even if the lesion clinically appears benign.
